# The utility of quantitative ^18^F-FDG PET/CT-derived parameters as prognostic factors for predicting overall survival in radioiodine-refractory differentiated thyroid cancer

**DOI:** 10.22038/aojnmb.2025.84029.1596

**Published:** 2025

**Authors:** Mai Hong Son, Nguyen Thi Phuong, Le Ngoc Ha

**Affiliations:** Department of Nuclear Medicine, Hospital 108, Hanoi, Vietnam

**Keywords:** Differentiated thyroid cancer Radioiodine-refractory, ^18^F-FDG PET/CT, ^18^F-FDG metabolic parameters, Overall survival

## Abstract

**Objective(s):**

This study investigates the relationship between quantitative ^18^F-FDG PET/CT metabolic parameters and overall survival (OS) in patients with radioiodine-refractory differentiated thyroid cancer (RAI-R DTC).

**Methods:**

We conducted a prospective analysis of 127 patients with RAI-R DTC. Quantitative metabolic parameters including SUV_max_, SUV_mean_, SUV_peak_, total metabolic tumor volume (MTV), and total lesion glycolysis (TLG) were assessed in ^18^F-FDG -avid recurrent or metastatic lesions via ^18^F-FDG PET/CT imaging. Patients were monitored for disease progression and mortality for at least one-year post PET/CT imaging. Receiver operating characteristic (ROC) curves were used to establish cut-off values for predicting 5-year mortality, while the Kaplan-Meier method estimated the 5-year survival rate. Univariate and multivariate Cox regression analyses identified prognostic factors associated with OS.

**Results:**

The metabolic parameters derived from ^18^F-FDG PET/CT demonstrated high sensitivity and specificity for predicting 5-year OS. ROC curve analysis established optimal cut-off values for SUV_max_ (20.27 g/mL), SUV_mean_ (7.46 g/mL), SUV_peak_ (7.8 g/mL), TLG (45.74 g/mL×cm³), and MTV (5.78 cm^3^) (AUC: 0.82, 0.78, 0.82, 0.82, and 0.86, respectively; p<0.001). Kaplan-Meier analysis revealed significantly lower OS in patients with higher values of these parameters compared to those with lower ones (survival rates: 42.1% vs. 95.6%, 65.5% vs. 96%, 52.3% vs. 96.3%, 46.5% vs. 97.3%, and 57.3 % vs. 98.3%, respectively; p<0.001). Univariate Cox regression identified SUV_max_, SUV_mean_, SUV_peak_, TLG, and MTV as significant predictors of 5-year OS (p<0.05). In multivariate analysis, SUV_peak_ and MTV emerged as independent predictors of OS.

**Conclusion:**

Quantitative ^18^F-FDG PET/CT-derived parameters are significant predictors of 5-year OS, exhibiting high sensitivity and specificity. Elevated values of these parameters correlate with increased mortality rates. Our findings suggest that SUV_peak_ and MTV are independent prognostic factors for 5-year OS in patients with radioiodine-refractory DTC.

## Introduction

 Differentiated thyroid carcinoma (DTC) generally has a favorable prognosis due to its slow progression and good response to radioiodine therapy (RAI). However, 5% to 15% of DTC cases and 50% of metastatic DTCs become refractory to RAI treatment (1-3). RAI-refractory (RAI-R) thyroid carcinoma cells result from the loss of differentiated features, including the inability to uptake iodine, leading to more aggressive behavior. Consequently, metastatic RAI-R DTC carries a much worse prognosis, with 10-year survival rates of only 10% (4). The systemic treatment options for RAI-R with metastases are limited and associated with serious side effects (5). 

 Therefore, identifying factors of poor prognosis is crucial for selecting appropriate treatment strategies to improve the clinical outcomes (6).

^18^F-FDG PET/CT is an effective modality in diagnosing recurrence and/or metastases in DTC patients with RAI-R. In patients with RAI-R DTC, tumor cells undergo dedifferentiation, losing their ability to concentrate iodine while simultaneously exhibiting increased glucose metabolism, making them more detectable on ^18^F-FDG PET (7). This phenomenon, known as the "flip-flop" effect, refers to the inverse relationship between RAI uptake and ^18^F-FDG PET avidity in DTC (8), (9). This shift reflects a more aggressive tumor phenotype and is often associated with resistance to RAI therapy (3). 

 Clinically, recognizing the ^18^F-FDG avid lesions is crucial for selecting appropriate imaging modalities and optimizing treatment strategies for metastatic or recurrent DTC (10). Beside location, number and size of malignant RAI-R lesions, ^18^F-FDG PET/CT provides the semi quantitative parameters derived from uptake measurement including standardized uptake values (SUVs) such as SUV_max_, SUV_mean_, SUV_peak_ and metabolic volume (MTV), total lesion glycolysis (TLG). The quantitative metabolic parameters were proposed to predict disease progression and prognosis of DTC patients with RAI-R (11). 

 Robin et al. reported a significant correlation between SUV_max_ and overall survival (OS) (12). However, the specific role of SUV_max_ in predicting prognosis remains a debate. Additionally, the number of ^18^F-FDG avid lesions and SUL_peak_ were identified as independent predictors of disease progression in a systematic review (11). Moreover, Poorni Manohar et al. revealed that high MTV and TLG were associated with high risk of death and those parameters can be consider as dynamic risk stratification factors to select appropriated follow-up or treatment strategies (13). 

 However, previous studies were retrospective with small patient cohorts and short follow-up periods. Furthermore, the measurement of quantitative ^18^F-FDG PET/CT parameters differs across PET centers because of the absence of standardized measurement methods, imaging protocols, and PET/CT systems (11). To the best of our knowledge, there have been limited studies investigating the role of ^18^F-FDG PET/CT-derived metabolic parameters in predicting OS with long-term follow-up of 5 and 10 years in patients with RAI-R. Therefore, this study aims to assess the relationship between ^18^F-FDG PET/CT-derived metabolic parameters and OS in post- operative metastatic DTC patients with RAI-R.

## Methods

### Study’s population

 A prospective study was conducted at the Department of Nuclear Medicine, Hospital 108, who underwent ^18^F-FDG PET/CT imaging from January 2016 to December 2022. The criteria for RAI-R was one of following conditions: 1) no initial RAI uptake, 2) lack of RAI uptake in at least one metastasis after treatment, 3) progression of at least one DTC recurrent metastasis despite RAI uptake in all metastases and extensive RAI exposure, or 4) DTC patients who have received a cumulative dose exceeding 600 mCi (22.2 GBq) without a treatment response (6). A total of 254 post-operative DTC patients with elevated thyroglobulin (Tg) levels >10 ng/mL and a negative whole-body iodine scan after RAI therapy underwent ^18^F-FDG PET/CT to detect recurrent or metastatic lesions. Among these patients, 127 (54%) had recurrent or metastatic lesions confirmed by ^18^F-FDG PET/CT findings which were validated by cytology, histopathology, or evidence of disease progression on follow-up imaging. The median age of the patients was 53.1 years, with a range from 17 to 79 years. Histopathology was classified according to the 2017 WHO criteria, with 88.2% of papillary, 11.8% of follicular and aggressive types. Post-operative TNM staging was assessed according to the AJCC 8th edition, and recurrence risk was stratified based on the 2015 ATA guidelines (10, 14). There were 98 (77.2%) of DTC patients in stage pT1-2 and 29 (22.8%) of DTC patients in stage pT3-4. Both serum thyroglobulin (Tg) and anti-Tg levels were measured using the Elecsys 2010 immunoassay analyzer (Roche, Switzerland) through an electrochemiluminescence immunoassay. Stimulated Tg was assessed after a 4-week withdrawal of thyroid hormone (levo-thyroxine), during which triiodothyronine was used for the first 2 weeks to stimulate TSH levels to exceed 30 µUI/ml (15). Thyroglobulin doubling time (Tg-DT) was estimated based on at least three consecutives unstimulated Tg before the ^18^F-FDG PET/CT scan, with suppressed TSH levels ≤0.5 µIU/mL. All recurrent and metastatic DTC patients with RAI-R who underwent ^18^F-FDG PET/CT were followed-up and treated according to 2015 ATA guidelines (10). Surgery, radiotherapy, systemic therapy, or active surveillance were selected for the most appropriate treatment for RAI-R DTC patients by the thyroid carcinoma board at Hospital 108. After treatment, patients underwent follow-up every 3–12 months, including clinical examination, serum Tg and anti-Tg antibody testing, and neck ultrasound to monitor for recurrence or progression. If no clinical symptoms, Tg elevation, or suspicious lymph nodes were detected, routine follow-up was continued. Suspected recurrence or progression warranted further evaluation with CT or ^18^F-FDG PET/CT. If confirmed, a multidisciplinary team determined the appropriate treatment approach. The study's endpoint was OS over a five-year period.

### 18F-FDG PET imaging

^18^F-FDG PET/CT scans were performed by the GE Discovery 710 system (GE Healthcare, Milwaukee, WI, USA) according to the European Association of Nuclear Medicine (EANM) operational guidelines when patients was under TSH suppression (16). Before scan, serum glucose levels were examined to exclude the patients with hyperglycemia. Each patient fasted at least 6-hour before PET/CT scan with a blood glucose level below 200 mg/dL. An intravenous injection of 2.5 MBq/kg body weight (±10%) of ^18^F-FDG was administered, and scans were performed approximately 60±10 minutes after the injection, covering the area from the skull base to the mid-thigh. The CT scan parameters included a voltage of 120 kV, a current of 100 mAs, a slice thickness of 5 mm, image reconstruction at 3.75 mm, and a pitch of 1.6. PET images were reconstructed using an iterative algorithm with CT-based attenuation correction. Whole-body PET images were obtained in three-dimensional mode, ranging 6-8 bed positions, with each position scanned for 1.5 minutes. The transaxial field of view (FOV) was 50 cm, and the images were reconstructed using an iterative algorithm with 20 subsets and 4 iterations, resulting in a matrix size of 256×256. ^18^F-FDG PET/CT images were reviewed in transaxial, sagittal, and coronal planes using the PET Onco Viewer on the GE workstation (version 4.7, GE Healthcare). Any focal tracer uptake that differed from the physiological background and showed higher activity than the surrounding tissue was considered indicative of disease. Two experienced nuclear medicine physicians with comprehensive knowledge of the patient's clinical history independently interpreted the ^18^F-FDG PET/CT results and the consensus was made.

### 18F-FDG PET/CT-derived parameters

 The volume of interest (VOI) was placed near physiological ^18^F-FDG-avid structures on attenuation-corrected PET images using the AW workstation version 4.7 (GE Healthcare, Milwaukee, WI, USA). The region of interest (ROI) within the lesions was then assessed on PET imaging with reference to CT. The volume of interest (VOI) was placed near physiological ^18^F-FDG-avid structures on attenuation-corrected PET images using the AW workstation version 4.7 (GE Healthcare, Milwaukee, WI, USA). Tumor volume was determined using iterative adaptive threshold segmentation provided by the vendor's software (PETVCAR, GE Healthcare). This algorithm employed a gradient vector slope method to establish a threshold value that distinguishes the tumor from surrounding tissues, with the SUV maximum value within the bounding box being adjusted by a 'w' factor (ranging from 0 to 1, with a default of 0.5). The tumor borders were automatically contoured, and the metabolic tumor volume (MTV) was calculated as the tumor volume. SUV_max_ and SUV_mean_ were defined as the maximum and average SUV values within the tumor volume, respectively. SUV_peak_ represented the average SUV within a 1 cm³ spherical region centered on the hottest point in the tumor. Total lesion glycolysis (TLG) was calculated by multiplying SUV_mean_ by MTV. The metrics for each lesion were recorded, followed by the calculation of the SUV_max_, SUV_mean_, and SUV_peak_ of the most active lesion, as well as the total MTV (tMTV) and total TLG (tTLG) across all ^18^F-FDG-avid metabolic lesions (17).

### Statistical Analysis

 Statistical Package for the Social Sciences (SPSS) version 25.0 (IBM Corp) was used for statistical analysis. Categorical variables were analyzed using the chi-square test or Fisher’s exact test. Continuous variables that followed a normal distribution were assessed with paired Student t-tests or repeated measures ANOVA, while variables not following a normal distribution were compared using the Mann–Whitney U test. The predictive ability of each ^18^F-FDG PET/CT parameter for OS was evaluated by determining the optimal cutoff value, sensitivity, and specificity through receiver-operating characteristic (ROC) curve analysis, with the diagnostic performance measured by the area under the curve (AUC). 

 Differences between the AUCs of the ROC curves were tested for statistical significance using the DeLong test. Significant predictors of OS were identified through logistic regression analysis based on the Cox model. The predictive value of clinical factors and ¹⁸F-FDG PET/CT parameters was initially assessed using univariate analysis. Variables with a significance level of p<0.05 were included in the multivariate analysis, which was conducted using the stepwise method. Estimation of OS was conducted using the Kaplan–Meier method, with a significance threshold of p<0.05.

## Results

**Figure 1:**
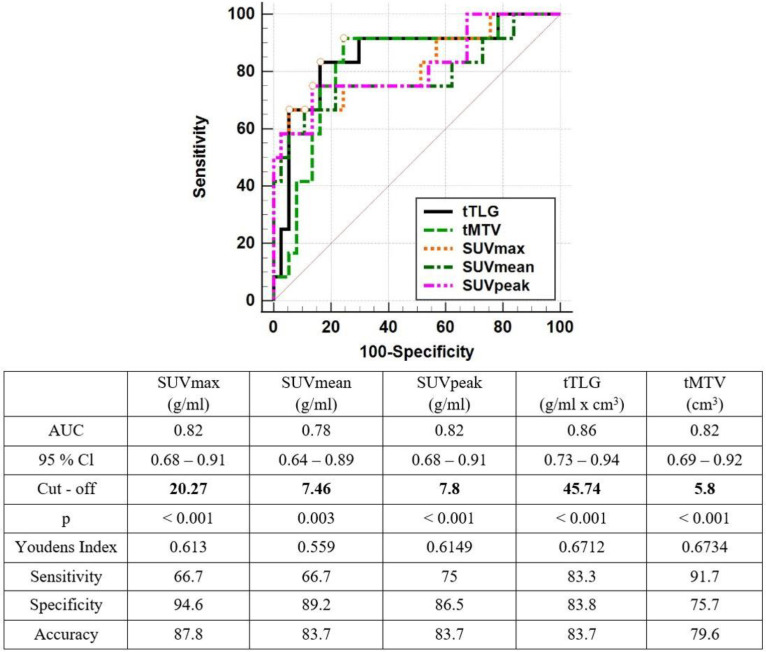
ROC curve analysis demonstrates the value of metabolic parameters derived from ^18^F-FDG PET/CT in predicting the five-year OS

**Figure 2:**
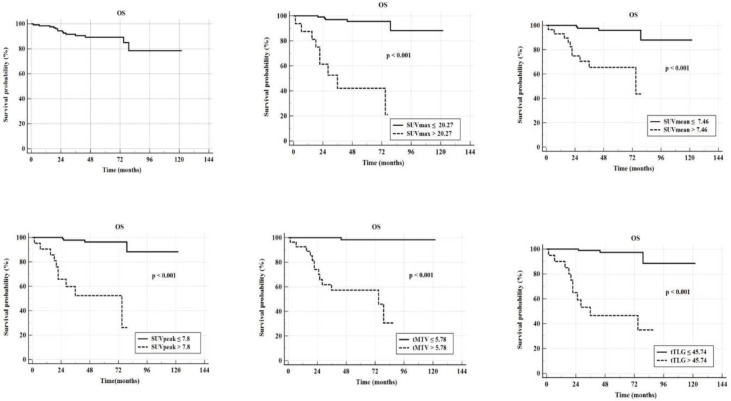
Kaplan-Meier curve illustrates the relationships between metabolic parameters derived from ^18^F-FDG PET/CT and the five-year OS

 General characteristics of DTC patients with RAI-R are illustrated in Table 1. The most common locations of metastatic lesions were the cervical lymph nodes (78%), lungs (23.6%), mediastinal lymph nodes (18.1%), and bones (9.4%). After stratification using PET/CT, all of patients treated with hormone therapy to suppress TSH below 0.1 µUI/mL. There were 59 out of 127 patients (46.5%) indicated for radical surgery, while 41 out of 127 patients were followed up. The median time for follow-up was 47.33 months (range 1.57-122.1). The mortality rate was 14 out of 127 (11%) with the death rates of 1.6%, 7.9% and 9.4% at 1 year, 3 years, and 5 years, respectively. The AUC of tTLG was 0.86, which was higher than those of tMTV, SUV_peak_, SUV_mean_, and SUV_max_. However, there was no significant difference between the AUCs of these metabolic parameters in predicting mortality over a five-year period (DeLong test, p>0.05, Figure 1). All metabolic parameters derived from ^18^F-FDG PET/CT showed a significant relationship to OS over the five-year period (p<0.001). The cutoffs for SUV_max_, SUV_mean_, SUV_peak_, tTLG, and tMTV were 20.27 g/ml, 7.46 g/ml, 7.8 g/ml, 45.74 g/ml×cm³, and 5.78 cm^3^, respectively, which were significant in predicting the five-year survival rate in RAI-R DTC patients after the ^18^F-FDG PET/CT scan (p<0.001, Figure 2).

 Patients with SUV_max_ >20.27, SUV_mean_ >7.46, SUV_peak_ >7.8, tMTV >5.78, and tTLG >45.74 exhibited significantly lower OS than the other group (p<0.001). The 5-year survival rates declined to 42.1% (95% CI: 18.1–65.9%), 65.5% (95% CI: 48.4–82.6%), 52.3% (95% CI: 28.7–75.9%), 57.3% (95% CI: 38.0–76.6%), and 46.5% (95% CI: 23.2–69.8%), respectively (Figure 2). Univariate analysis revealed that age >55, follicular or aggressive histopathologic types, cumulative ^131^I dose >600 mCi, stimulated Tg >168.1 ng/ml, Tg-DT ≤12 months, lung metastases, bone metastasis, SUV_max_ >20.27 g/ml, SUV_mean_ >7.46 g/ml, SUV_peak_ >7.8 g/ml, tTLG >45.74 g/ml×cm³, and tMTV >5.78 cm^3^ were factors in predicting survival (p<0.05). However, in multivariate analysis, only Tg-DT ≤12 months, SUV_peak_ >7.8 g/ml and tMTV >5.78 cm^3^ were identified as prognostic factors for predicting OS, with HR=8.34, p=0.011; HR=5.2, p=0.021 and HR=13.1, p=0.025, respectively (Table 2).

**Figure 3:**
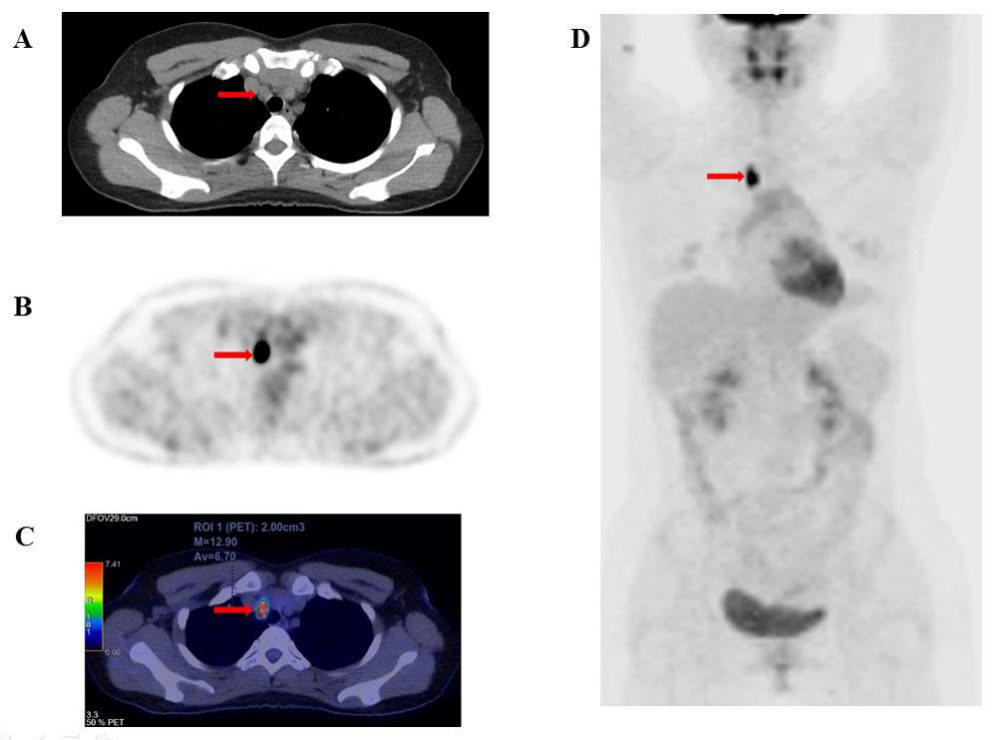
A 29-year-old female patient diagnosed with papillary thyroid carcinoma (pT1N1bM0) underwent total thyroidectomy and neck dissection. She received radioactive iodine (RAI) therapy with a cumulative ¹³¹I dose of 400 mCi, achieving a stimulated thyroglobulin (Tg) level of 171.8 ng/mL and a negative Tg doubling time (Tg-DT). PET/CT imaging revealed a lesion in the left cervical lymph node (group VII, arrow). The semi-quantitative metabolic parameters were as follows: SUV_max_: 12.9, SUV_mean_: 6.7, SUV_peak_: 7.74, tMTV: 2 cm³, and tTLG: 13.43 g/mL × cm³. The patient subsequently underwent a group VII cervical lymph node dissection, achieving complete disease remission postoperatively

**Figure 4:**
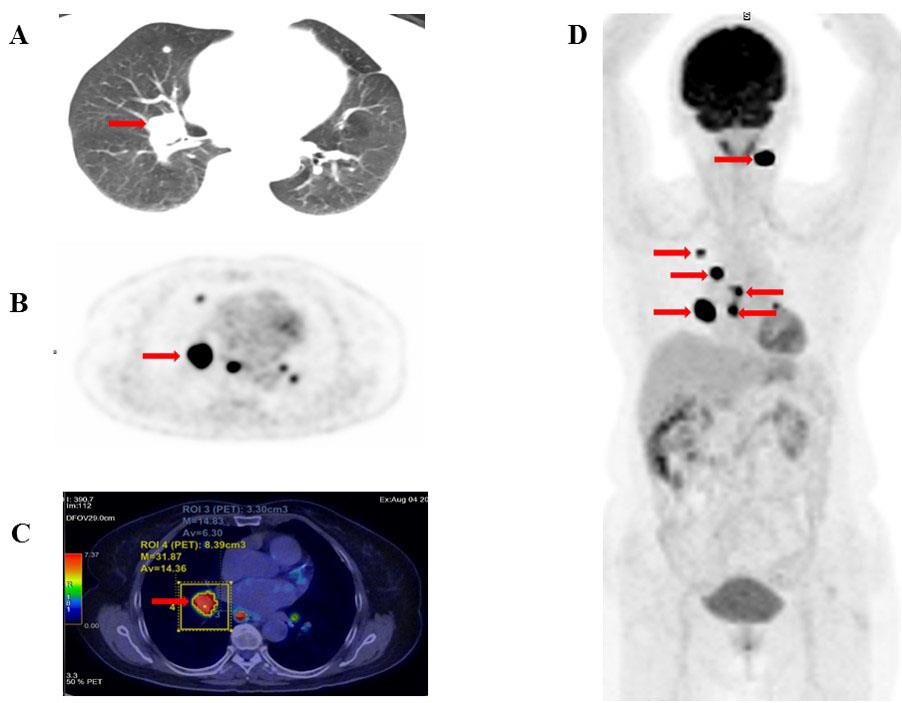
A 72-year-old female patient diagnosed with papillary thyroid carcinoma (pT1N1bM) underwent total thyroidectomy and neck dissection. She received radioactive iodine (RAI) therapy with a cumulative ¹³¹I dose of 300 mCi, achieving a stimulated thyroglobulin (Tg) level of 226.5 ng/mL and a Tg doubling time (Tg-DT) of 9.9 months. PET/CT imaging revealed metastatic lesions in the cervical lymph nodes, lungs, and mediastinal lymph nodes. The semi-quantitative metabolic parameters were as follows: SUV_max_: 39.42, SUV_mean_: 14.36, SUV_peak_: 25.52, tMTV: 20.8 cm³, and tTLG: 240.4 g/mL × cm³. The patient was treated with Sorafenib. Disease progression occurred 20.3 months after the PET/CT scan, and the patient passed away 74 months after diagnosis

Figure 3 (A. CT, B. PET, C. fused PET/CT, D. maximum intensity projection) illustrates a representative case of a single lesion detected by ^18^F-FDG PET/CT, which was associated with a good prognosis. Conversely, Figure 4 (A. CT, B. PET, C. fused PET/CT, D. maximum intensity projection) shows a case of multiple lesions in multiple organs detected by ^18^F-FDG PET/CT, indicating a poorer prognosis.

**Table 1:** General characteristics of DTC patients with RAI-R

			Number (n=127)	Percent (%)
Age (year)	<55		63	49.6
	≥55		64	50.4
	Median (range)		53.1 (17–79)	
Gender	Male		21	16.5
	Female		106	83.5
	Male/female ratio		1/5.05	
Histopathologic types	Papillary		112	88.2
	Follicular/aggressive		15	11.8
AJCC8th stage	I - II		98	77.2
	III - IV		29	22.8
Risk of recurrence (ATA 2015)	Low – intermediate		83	65.4
	High		44	34.6
Accumulative dose of ^131^I (mCi)	≤600		110	86.6
	>600		17	13.4
	Median, range		403 (80–1300)	
Number of ^131^I therapy (Mean±SD, range)			2.96±1.39 (1–8)	
Tg and Tg-DT prior to PET/CT (ng/ml)	Stimulated Tg (median, range)		168.1 (10.7–5982)	
	Tg – DT	>12 months or negative	90	70.9
		≤12	37	29.1
Metabolic parameters derived from ^18^F-FDG PET/CT (median, IQR)	SUV_max_ (g/ml)		6.02 (3.16–11.35)	
	SUV_mean_ (g/ml)		3.66 (2.34–6.0)	
	SUV_peak_ (g/ml)		3.06 (1.82–7.01)	
	tTLG (g/ml×cm^3^)		3.76 (0.85–23.35)	
	tMTV (cm^3^)		1.23 (0.3–4.95)	
Time follow-up after ^18^F-FDG PET/CT scan (median, range, month)			47.33 (1.57–122.1)	

**Table 2:** Univariate and multivariate Cox analysis of clinicopathological and metabolic parameters for predicting five-year OS

Factors	Univariate			Multivariate		
	HR	95% CI	p	HR	95% CI	p
Age ≥55	6.3	1.4 – 28.2	0.016	-	-	-
Male	0.3	0.04 – 2.35	0.252	-	-	-
Follicular or aggressive histopathologic type	5.6	1.9 – 16.3	0.002	-	-	-
Stage III – IV (AJCC 8)	1.04	0.32 – 3.37	0.95	-	-	-
High risk of recurrence	0.81	0.27 – 2.46	0.71	-	-	-
Accumulative dose of 131I >600 mCi	3.41	1.14 – 10.2	0.028	-	-	-
Stimulated Tg >168.1 ng/ml	5.49	1.2 – 24.7	0.026	-	-	-
Tg – DT ≤12 months	17.55	3.9 – 78.6	<0.001	8.34	1.62 – 43	0.011
Local recurrence	0.7	0.2 – 3.1	0.621	-	-	-
Cervical lymph node metastasis	0.43	0.15 – 1.25	0.121	-	-	-
Lung metastasis	3.2	1.1 – 9.1	0.031	-	-	-
Bone metastasis	8.8	3.04 – 25.5	<0.001	-	-	-
SUV_max_ >20.27 (g/ml)	24.1	7.3 – 79	<0.001	-	-	-
SUV_mean_ >7.46 (g/ml)	12	3.7 – 38.8	<0.001	-	-	-
SUV_peak _>7.8 (g/ml)	18.7	5.8 – 60.2	<0.001	5.2	1.3 – 21.3	0.021
tTLG >45.74 (g/ml×cm^3^)	25.4	7.1 – 91.7	<0.001	-	-	-
tMTV >5.78 (cm^3^)	55.6	7.3 - 426	<0.001	13.1	1.4 – 125.4	0.025

## Discussion

Our study was conducted on 122 patients with differentiated thyroid cancer (DTC) who had recurrent or metastatic radioiodine-refractory (RAI-R) lesions detected on ^18^F-FDG PET/CT, with a median follow-up time of 40 months (range: 1.57–82.5 months). The prognostic role of classical clinical factors including age, histopathological type, extension of the primary tumor, disease stage, distant metastases were confirmed in previous studies (18). In addition, our study showed that quantitative metabolic parameters derived from ^18^F-FDG PET/CT provided significant prognostic implication for predicting 5-year OS with specificity from 75.7 % to 94.6 %. The Kaplan-Meier curve, based on thresholds determined from the ROC curve, shows that patients with higher ^18^F-FDG PET/CT metabolic parameter values have shorter OS. Specially, SUV_peak_ >7.8 g/ml and tMTV >5.78 cm³ were identified as independent predictors of five-year OS. Most other studies have similarly highlighted the association of ^18^F-FDG PET/CT with disease progression and survival in patients with DTC (11).

 The standardized uptake value (SUV) represents the level of glucose metabolism in tumors with malignant lesions typically exhibiting higher values. Numerous studies have shown that tumors with elevated FDG uptake are often associated with more aggressive histological subtypes, reflecting a progression toward higher malignancy compared to the primary tumor (19). In our study, metabolic parameters including SUV_max_, SUV_mean_, and SUV_peak_ were found to correlate with 5-year OS, with predictive thresholds of 20.27 g/ml, 7.46 g/ml and 7.8 g/ml, respectively. Deandreis D. et al. (2011) demonstrated that SUV_max_ >10 was significantly associated with OS in distant metastatic RAI-R DTC (p=0.02) (20). Similarly, Masson-Deshayes S. et al. (2015) reported that SUV_max_ >10 and SUL_peak_ >5 were prognostic factors for OS, though neither was identified as an independent predictor (21). However, SUVs are often limited to a single lesion on PET/CT, which may not represent the metabolic characteristics of all tumor lesions. Moreover, these values can be influenced by patient body weight, uptake time, administered ^18^F-FDG dose, and the acquisition and reconstruction protocols of different scanners and software. As a result, relying solely on SUVs for OS prediction has limitations. To address these challenges, quantitative metrics such as MTV and TLG have implemented in recent years. These metrics have demonstrated significant potential in assessing treatment response and predicting OS across various types of cancer (22, 23). Manohar et al. demonstrated the prognostic value of MTV >9.08 ml and TLG >49.1 in predicting OS in a cohort of 62 RAI-R DTC patients (13). Another study by Domenico et al. (2021) involving 87 patients with RAI-R DTC and elevated serum Tg but negative ^131^I scintigraphy showed that tMTV and tTLG were independent predictors of OS. These findings highlighted the critical role of whole-tumor quantitative metrics in improvement of predicting clinical outcomes in RAI-R DTC (24). 

 In our study, tMTV and tTLG are predictive factors for 5-year OS in patients with RAI-R DTC. Interstingly, tMTV was proved as the independent prognostic factor in multivariate analysis (HR of 55.6; 95% CI: 7.3–426; p<0.001). Therefore, patients with high tMTV values require active surrvilance and tailored appropriate treatment strategies. However, the threshold values for TLG and MTV differ across studies due to variations in patient populations and measurement methods at each center. Therefore, each center should establish its own threshold for predicting mortality risk in patients with RAI-R DTC.

 In our study, iterative adaptive threshold segmentation (IATS) has emerged as a robust method for delineating metabolic tumor volumes (MTV) in FDG PET/CT imaging. Its accuracy is primarily focused on an iterative refinement process that adjusts segmentation thresholds based on local image characteristics, thereby minimizing the impact of variations in uptake intensity and background noise. This dynamic adjustment enhances the accuracy of tumor boundary identification, especially in heterogeneous lesions (25). In contrast, fixed thresholding methods apply a predetermined percentage of the maximum standardized uptake value (SUV_max_) to define lesion boundaries (e.g., 41% SUV_max_) (26). However, this method lacks adaptability to tumor heterogeneity and background noise, often leading to over- or under-segmentation. Gradient-Based Methods identifies edges based on intensity gradients in the PET image. Although they improve contour definition, they can be sensitive to noise and require additional pre-processing (27). More recently, deep learning and radiomics-based segmentation techniques have leveraged large datasets to improve accuracy (28). Nevertheless, this modern method of segmentation requires extensive training data and computational resources (29). Compared to other techniques, IATS provides a balance between adaptability and computational efficiency. The improvement of its accuracy is reducing user dependency and adjusting thresholds dynamically, leading to more reliable MTV quantification in small tumor volume of thyroid carcinoma. Additionally, IATS mitigates the limitations of static thresholding while avoiding the high complexity of deep learning models. Moreover, in our center the EARL accreditation was implemented to standardize the performance of scanners and minimize the influence of technical factors that contribute to SUV variability (30). A key strength of our study is the use of ROC curve analysis to identify the best compromise between sensitivity and specificity, thereby establishing the cut-off values specific to our center.

 However, this study has some limitations. The relatively short average follow-up period of 40 months may be insufficient to fully evaluate OS, particularly given the slow progression of recurrent or metastatic RAI-R DTC. Furthermore, variations in the extent of metastatic lesions and treatments received among patients may impact the nature of disease.

## Conclusion

 Metabolic parameters derived from ^18^F-FDG PET/CT demonstrate high sensitivity and specificity in predicting five-year OS in DTC patients with RAI-R. Higher values of these parameters are associated with worse mortality rates. Our findings suggest that quantitative parameter, including SUV_peak_ and tMTV serve as independent prognostic factors for five-year OS in RAI-R DTC patients.
